# Impact of COVID-19 pandemic on family planning and sexual transmitted infection services in Thailand: results from WHO survey

**DOI:** 10.1186/s12978-025-02092-0

**Published:** 2025-09-30

**Authors:** Jen Sothornwit, Pisake Lumbiganon, Nampet Jampathong, Somporn Rungreangkulkij, Netchanok Kaewjanta, Caron Kim, Moazzam Ali, WHO HRP Social Science Research Team, Luis Bahamondes, Jose Guilherme Cecatti, Vilma Zotareli, Rachel E Soeiro, Karayna G Fernandes, Mariana B Rogerio, Samira M Haddad, Silvana F Bento, Karla S Padua, Aline Munezero, Charles M Charles, Montas Laporte, Eunice Chomi, Seni Kouanda, Flore Marie Gisèle Donessouné, Armel Sogo, Kun Tang, Yueping Guo, Hanxiyue Zhang, Yifan Zhu, Ge Yang, Chunxiao Peng, Xizhuo Xie, Hao Wang, Deda Ogum Alangea, Kwasi Torpey, Emefa Judith Modey, Adom Manu, Ernest T. Maya, Rozina Karmaliani, Laila Ladak, Arusa Lakhani, Marina Baig, Yasmin Parpio, Salima Somani, Pisake Lumbiganon, Jen Sothornwit, Nampet Jampathong, Somporn Rungreangkulkij, Marleen Temmerman, Ferdinand Okwaro, Abdu Mohiddin, Massimo Mirandola, Maddalena Cordioli, Alessia Savoldi, Simone Garzon, Stefano Uccella, Nigel Sherriff, Alexandra Sawyer, Catherine Aicken, Jörg W Huber, Jaime Vera, Deborah Williams, Moazzam Ali, Caron Kim, Vanessa Brizuela, Hamsadvani Kuganantham, Grace Kapustianyk, Igor Toskin, Soe Soe Thwin, Armando Seuc, Gabriela Garcia Camacho

**Affiliations:** 1 Department of Obstetrics and Gynecology, Faculty of Medicine, Khon Kaen University, Mueang Khon Kaen District, Khon Kaen, 40002 Thailand; 2 Cochrane Thailand, Khon Kaen University, Khon Kaen, Thailand; 3 Department of Psychiatric Nursing, Faculty of Nursing, Khon Kaen University, Khon Kaen, Thailand; 4 Department of Sexual and Reproductive Health and Research, World Health Organization, Geneva, Switzerland

**Keywords:** COVID-19, Public health, Reproductive medicine

## Abstract

**Background:**

COVID-19 pandemic has put tremendous burden on health services. Only limited evidence, however, is available to identify the impact of COVID-19 on sexual and reproductive health (SRH). This world health organization (WHO)-led research sought to evaluate health systems focusing on SRH in Brazil, Burkina Faso, China, Ghana, Italy, Pakistan, Thailand, and the United Kingdom.

**Methods:**

The study was conducted in Thailand on two levels using a mixed-methods design: 1) Individual level included in-depth interviews and focus group discussions with clients (and their partners, where applicable) and healthcare providers (HCPs) to investigate service perceptions and obstacles to SRH service utilization; 2) health facility level, a quantitative evaluation of health facility preparedness for SRH service provision was performed using an adapted version of the WHO Service Availability and Readiness Assessment (SARA) tool. The data was collected at two timepoints, baseline and endline, at time intervals of 9 months.

**Results:**

Almost all SRH services were maintained with some shortage of supply such as medications for safe abortion during the first few months. Both providers and clients perceived that all SRH services should be maintained. Some clients were concerned about fear of getting COVID-19 infection while visiting the facility. Some clients switched from short-acting to long-acting contraceptive methods. At the endline, this affect was less obvious since a large proportion of clients were familiar with the pandemic and already received vaccination.

The Centre for COVID-19 Situation Administration (CCSA) was established to update COVID-19 pandemic situation, new government policy and intervention to reduce fake news. Telemedicine was used to reduce avoidable appointments. For postpartum women, appointment tended to be more individualized. For those who required pregnancy protection, contraceptive methods were offered to clients before discharge from the hospital. A follow-up visit was performed using both telemedicine and in-person visit at the hospital. For those required medications such as antibiotics for STI, home delivery to clients was provided.

**Conclusions:**

This study demonstrated that COVID-19 pandemic had some but non-significant effect on SRH services. The two major referral hospitals in Northeast, Thailand had service readiness to provide SRH services during the COVID-19 pandemic and pandemic recovery.

## Background

In 2002, Thailand initiated the implementation of a comprehensive healthcare program known as Universal Health Coverage (UHC), which ensures that all Thai citizens are granted access to a wide array of essential healthcare services encompassing health promotion, disease prevention, medical treatment, rehabilitation, and palliative care [1]. Concomitantly, the National Health Security Office (NHSO) was established in the same year. Its inception was rooted in the recognition of the necessity for effective and knowledge-driven management of public resources. The NHSO is unwaveringly committed to contributing to the nation's sustainable development, with an overarching emphasis on advancing the common good as its paramount objective. The NHSO is responsible for overseeing all preventive and health promotion services, including sexual and reproductive health services, for the entire population. The NHSO's program for promoting health and preventing diseases has initiated a campaign to offer free access to oral contraceptive pills (OCPs) and condoms to all Thai individuals aged 15 and older. Additionally, in an effort to reduce teenage pregnancies, long-acting reversible contraception (LARC) options such as contraceptive implants and intrauterine devices are also made available to teenage women at no expense [2].

Thailand has recorded a total of 4,628,035 confirmed cases of COVID-19 and approximately 34,000 reported deaths so far. The first confirmed case in Thailand was documented on January 13th, 2020, with the first instance of local transmission reported by the end of that same month. The number of cases sharply increased in mid-March, reaching its peak on April 25, 2020. Subsequently, there was a gradual decline in new cases. Thailand experienced two major waves of outbreaks in June 2021 and December 2021, followed by another in January 2022 to May 2022. To combat the spread of the virus, various preventive measures were put in place, including the sudden closure of businesses in Bangkok and several other provinces, the suspension of international flights, the implementation of lockdown measures, state-mandated quarantine, and the promotion of social distancing. Additionally, the Centre for COVID-19 Situation Administration (CCSA) was established to provide the public with information regarding the current state of the disease as well as government policy and interventions [3, 4].

The COVID-19 pandemic limit the availability of sexual and reproductive health (SRH) services, leading to higher instances of unintended pregnancies, pregnancy-related complications, unsafe abortions, intimate partner violence, and elevated levels of maternal and infant mortality [5]. This world health organization (WHO)-led research sought to evaluate health systems focusing on SRH in Brazil, Burkina Faso, China, Ghana, Italy, Pakistan, Thailand, and the United Kingdom. The objectives of this research are to evaluate the accessibility and standard of SRH services focusing on contraceptive and sexual transmitted infection (STI), as well as the impediments hindering the utilization of these services, and post-pandemic recovery as perceived by both clients and providers, within Thailand during the COVID-19 pandemic [6].

## Methods

### Study design

This research employs a repeated cross-sectional design, employing a combination of quantitative and qualitative research approaches. The study involves two distinct data collection instances, including the baseline and end-line assessments, which were separated by a period of 9 months. The primary objective of incorporating these two data collection points was to capture and disseminate empirical insights pertaining to SRH services within the local context at the baseline assessment. Subsequently, at the end-line assessment, the purpose was to monitor and illustrate the alterations and enhancements observed in these services over the specified time frame.

### Study setting

The study was conducted in two health facilities in Khon Kaen Province, northeast region of Thailand. These included Srinagarind Hospital, Khon Kaen University, which is a major teaching and referral hospital and Khon Kaen Hospital, which is a major referral hospital under the Ministry of Public Health in upper northeast Thailand. Both hospitals took care of almost all COVID-19 patients in Khon Kaen Province. The majority of the COVID-19 cases in Khon Kaen would be admitted in these two hospitals, following the policy of provincial health office.

### Study population

The participants consisted of healthcare providers, women seeking SRH services at selected hospitals, as well as their accompanying partners.

### Qualitative methods

#### Participants

We conducted in-depth interviews (IDIs) with providers who were most knowledgeable about readiness and availability of SRH services, reproductive age women (18–49 years) who have sought to receive reproductive health services (contraception, or STI) and their partners. Participants for the focus group discussion (FGD) were women and the men accompanying the women to the hospitals. Interviews were done at two different time points: the baseline in December 2021to February 2022 and the endline in June 2022 to August 2022.

#### Sampling, recruitment and informed consent

Purposive sampling was used. We planned to recruit at least 10 women and six men. Health care providers were purposively selected to participate in the IDIs and in the health facility assessment. We expected 2–3 providers to participate in IDI for each facility. Participants were recruited until the information reached saturation. Further details are provided in the text which follows.

#### Data collection and data management

After obtaining written informed consent, qualified researcher conducted FGD and IDIs with clients to investigate the psychosocial impact of COVID-19 on various aspects, including fertility desires, knowledge about COVID-19, risk perceptions, concerns related to sexual and reproductive health (SRH), healthcare-seeking behaviors, experiences, barriers in accessing SRH services, reproductive health services like comprehensive abortion, prevention and treatment of sexually transmitted infections (STIs), and gender-based violence (GBV) care and support. However, this report focused on two main important issues including contraception and STIs. The participants also shared their specific informational and service-related needs in the realm of reproductive health.

For healthcare providers, IDIs were conducted using the World Health Organization's (WHO) six building blocks framework, with a particular emphasis on the provision of contraception, and STI prevention and treatment during the COVID-19 pandemic. The interviews were semi-structured and adhered to a predefined topic guide. Audio recordings of the interviews were made and subsequently transcribed verbatim.

#### Analysis

The analysis of all IDIs and FGDs adhered to a standard content analysis method. This entailed reviewing the word-for-word transcripts and notes to comprehend the participants'expressions, documenting emerging themes and data patterns, and summarizing the information. Codes were applied to create categories, and these categories were further distilled into themes that encapsulated the data's significance. We compared the findings between endline and baseline.

### Quantitative methods

We conducted a panel survey between September 2021 and May 2022 using questionnaire with two sections, general and facility assessment survey. The general section was completed by the clients who seek services at the time of data collection.

The evaluation of the readiness and availability of sexual and reproductive health (SRH) services in healthcare facilities drew inspiration from the WHO/USAID Service Availability and Readiness Assessment (SARA) tool which was described in detail in the published protocol [6].

#### Selection of clients

The anticipated monthly client count for both facilities was 250. We assumed that 10% of these clients would seek services related to sexual and reproductive health (e.g., family planning, sexually transmitted infection treatment, abortion care, etc.), with an acceptable margin of error of 5%. Consequently, we determined that a sample size of 89 clients per month was necessary. To estimate the percentage of clients receiving SRH care each month for a span of nine months, a total of 801 clients needed to be interviewed. Data collection occurred continuously, and ultimately, we successfully recruited a total of 893 participants, with 416 from Srinagarind Hospital and 477 from Khon Kaen Hospital.

#### Selection of healthcare providers

A single healthcare provider, chosen for their expertise in SRH services at the health facility, was designated to participate in the assessment. This individual held a senior position, either as a senior healthcare provider or administrator, for each segment of the questionnaire.

#### Data collection and data management

The data was double entered into the"OpenClinica"system by two distinct data operators: one for initial entry and the other for double-checking accuracy. Stringent measures were implemented to guarantee the data's quality. Every form underwent thorough inspections for completeness, logical errors, and unclear or irrelevant responses.

#### Analysis

Descriptive analysis was used to illustrate the basic characteristics of the different facilities, including the monthly number of clients, types of procedures provided, number of medical staffs, stocks of drugs. The data was presented as number and percentage. As Thailand faced many waves of the outbreak, therefore we could not compare between baseline and endline.

### Ethical consideration

Approval for this study, in accordance with ethical standards, was granted by the WHO Scientific and Ethics Review Committee under protocol ID CERC.0103. We received the approval (HE641001) from The Khon Kaen University Ethics Committee in Human Research on February 2021 and Khon Kaen Hospital Institute Review Board in Human Research (KEMOU64006) on March 2021.

## Results

### Qualitative component

Four FGDs were conducted at baseline (December 2021to February 2022). For endline, 2 FGDs were done (June 2022 to August 2022). IDIs with 6 providers, 20 female clients, and 19 male clients were conducted at baseline. For endline, IDIs with 3 providers, 20 female clients, and 17 male clients were conducted. Mean-age of the clients were 26.8 and 26.7 years for baseline and endline, respectively. While mean-age of the providers were 45.3 and 47.3 years for baseline and endline, respectively (Table 1).

**Table 1:** Characteristics of clients in baseline and endline participated in the interviews

Characteristics	Baseline (*n* = 39)	Endline (*n* = 37)
Mean age (year)	26.79 ± 7.68	26.7 ± 6.14
Sex		
Male	19 (48.7)	17 (45.9)
Female	20 (51.3)	20 (54.1)
Number of children		
0	26 (66.7)	12 (32.4)
1	7 (17.9)	17 (45.9)
2	5 (12.8)	8 (21.6)
3	1 (2.6)	0 (0)
Educational level		
Primary school	0 (0)	2 (5.4)
Secondary school	9 (23.1)	10 (27)
High school	11 (28.2)	12 (32.4)
Vocational training	6 (15.4	2 (5.4)
Bachelor’s degree and higher	13 (303.4)	9 (24.3)
Other	0 (0)	2 (5.4)
Marital status		
Single	10 (25.6)	12 (32.4)
Married	29 (74.4)	25 (67.6)
Occupation		
No	4 (10.3)	4 (10.8)
Student	3 (7.7)	4 (10.8)
Government officer	3 (7.7)	1 (2.7)
Employee	23 (60.0)	18 (48.6)
Self employed	5 (12.8)	9 (24.3)
Farmer	1 (2.6)	1 (2.7)
Monthly income (Baht)		
No	1 (2.6)	1 (2.7)
Uncertain	3 (7.7)	2 (5.4)
< 5,000	16 (41)	2 (5.4)
5,000–10,000	15 (38.5)	17 (45.9)
10,001–20,000	4 (10.3)	13 (35.1)
> 20,000	0 (0)	2 (5.4)
Religion		
Christianity	3 (7.7)	0 (0)
Buddhist	26 (92.3)	36 (97.3)
Other	0 (0)	1 (2.7)
Living community		
Rural	6 (15.4)	13 (35.1)
Semi-urban, semi-rural	12 (30.8)	6 (16.2)
Urban	21 (53.8)	18 (48.6)

Data present as mean ± standard deviation or n (%)

### Contraception services

#### Healthcare providers’ perception

- Perceived changes in the demand for contraception services during COVID-19 pandemic

Providers perceived that clients expressed more interest in birth control. Furthermore, they switched to a longer-lasting form of birth control.

“More needs (contraception) would be among students and children younger than 20 years. Moreover, I noticed more concern in postpartum women during pandemic. Previously, if you give advice, sometimes they might not want to use contraception. But now, everyone is more concerned……Because they don't want to get pregnant during the pandemic.” ………………Provider3kkhb.

“They (the clients) wanted to use birth control for a longer time. It turned out that they didn't want to come to the hospital often. From injectables, they switched to implants.” ………………Provider3kkhb.

- Availability of contraception services during COVID-19 pandemic

There was no shortage of contraceptive supply.

- Delivery of contraception services during COVID-19 pandemic

Providers of both facilities postponed appointments for non-urgent clients or advised them to receive services from a hospital nearby. For those who came, all clients had to be screened for COVID-19 before receiving service. During COVID-19 pandemic, postpartum women were provided with contraception options before their discharge, and nurses in the postpartum ward underwent training to counsel and facilitate the insertion of contraceptive implants promptly.

“During the first wave of outbreak, we called to postpone appointments for 2 weeks because we didn't know what the situation was at that time…… And if anyone was convenient to go to the nearby hospitals, we recommended them to do so.” ………………Provider3kkub

“We're responsible for providing the initial training. Before the pandemic, we didn't carry out implant insertions in the ward, correct? We're planning to educate the nurses on assisting the doctors in this process. The doctors will handle the actual procedure, and our role is to support them. Additionally, I've created a QR code to share information about postpartum care and different birth control methods.” ………………Provider3kkub

Telemedicine services via mobile phone was used as an innovation as a result of adapting to the pandemic to decrease in-person visit and promote social distancing policy. Timing for visit was individualized.

“We made a call instead. Normally after giving birth, we schedule a checkup at 6 weeks, right? But we called around 5 weeks instead. If they had problems, we advised them to come to the hospital within a week. But if they didn’t, we asked them to come within 3 months after delivery. We also provided contraception before discharge for those who expressed interest.” ………………Provider3kkub

For those who received online-visit and required medication, home-deliver medicine was also provided.

“During that time, there was a service for delivering medicine to patient’s home. …. sending medicines by logistics.” ………………Provider3kkub

- Difference between baseline and endline

During the six-month data collection period, we could not specify the date for post-COVID-19 pandemic (endline). During this period, no differences were observed in either the number of visits or contraceptive methods as well as the pattern of services for COVID-19.

#### Clients’ perspective

The following information was obtained from two hospitals. There might be some differences in the contextual issues between these two hospitals.

- Contraceptive method used, changes to contraceptive use, and pregnancy intention

There were three types of decision in choosing a birth control method, depending on the family context, including women themselves, women decided together with their partners or women made decisions together with their boyfriends and mothers. Both male and female clients intended to delay pregnancy due to two main reasons, fear of COVID-19 infection to the newborn and financial reason. Women stated that they opted for extended-acting methods because it reduced the frequency of hospital visits.

“I still don't want to get pregnant during COVID. …. I’m afraid of getting infected. I'm afraid I'll get COVID. Then the child will be abnormal.” ……………F5kkhe_5

- Finding out information about the service and how to access it

There were four main sources of birth control information including the hospital's Line App, the internet, healthcare providers, and from others such as friend.

“Listen to advice from friends who got the implant before. So, I chose to use it as well” ……………..F7kkhe

- ‘Patient journey’ – process of seeking and receiving care

Service at the family planning unit was convenient and fast. No need to sit and wait in line. The hospital had a strict COVID-19 prevention system.

“The service was good and it's open and not crowded. And it was very convenient. And the doctor was okay.” …………………M3kkue_3

- Perception of risk of (or safety from) Covid-19 infection during care-seeking

Clients were aware of the risk of COVID-19 infection when coming to the hospital because it was crowded.

“There is a risk because the number of people using the service is still quite large.” ……………..F7kkhe_7

- Barriers and facilitators to receiving care (including reasons for delays)

Some respondents perceived barriers including inconvenience of parking, postponement of appointments, overcrowded and long queues in hospital. Registration through the online system makes it convenient to not have to wait in line at the medical records unit, hence considered as facilitator.

“It's crowded. You might have to line up for a long time and wait for a long time. Even in the COVID-19 pandemic period, there were still a lot of people using the service” ……….F1kkhb

- Viewpoint and experience of clients on contraception service comparing between baseline and endline.

Issues with no difference include knowledge about COVID-19, services from providers, and barriers during getting services. Issues with difference include anxiety and concern declined between the baseline and endline, likely attributable to increased COVID-19 vaccination rates and familiarity with the ongoing pandemic.

### Detection, treatment and prevention of sexually transmitted infections

#### Healthcare providers’ perception

- Perceived changes in the demand for testing, care and treatment for STI during Covid-19 pandemic

During COVID-19 pandemic, there has been a decrease in people seeking services regarding STI.

“In the early days of the pandemic, it decreased a lot. Patients were worried about the pandemic situation. They were afraid of coming to the hospital and getting infected. The next thing was that their children or grandchildren didn't want them to come. Or they were unable to come due to the lockdown policy” …………….Provider1kkub

- Availability of STI testing, care, and treatment during COVID-19 pandemic – including health promotion and partner notification

There was no interruption to this service. Used more telephone for notification of examination results was employed to sustain the service.

“We also provided telemedicine services in some cases where it was not necessary to come. Or we delivered medicine to patient home to prevent the patients from flowing in from high-risk areas.” …………….Provider1kkub

If the individual receiving telemedicine requires medication, the hospital also offers a service for delivering medicines to their home.

“When it started, it was as if the patients were decreased. Patients were afraid. And we ourselves were afraid too. We had measures. That was, for non-emergency patients, we would deliver medicine to them at home.” ………………Provider3kkhb

- Difference between baseline and endline

No differences were observed. Providers were continuously trained in knowledge about COVID-19 prevention and screening before examining every client.

#### Clients’ perspective

- Needs related to STI services, finding out information about the service and how to access it

There was no clear information on the impact of pandemic on STI.

- Barriers and facilitators to receiving care (including reasons for delays)

Barriers to accessing services involve societal stigmatization of individuals with HIV, branding them as morally questionable, naïve, or promiscuous, leading to feelings of shame and reluctance to seek assistance. For facilitators, factor that promote service utilization include the convenience of available services.

“Yes (when I had a sexually transmitted infection) and I didn't dare to tell my friends, I was embarrassed and didn't know where to go. Didn't go for treatment.” ……………F4kkhb

“Now it's as if it's been managed to prevent crowding. I feel like it's better than before pandemic. According to my feelings, when I arrived, I could register. I don't have to sit and wait, sometimes for an hour or two” ……………F9kkub

- Psychosocial impact of the pandemic

Some clients mentioned that they were experiencing stress and facing financial difficulties.

“I feel overwhelmed as my financial resources fall short to cover my expenses.” ……………….F3kkue_3

- Viewpoint and experience of clients on STI service comparing between baseline and endline.

No difference was observed.

There was limitation for some themes since information retrieved from the interview of the clients who came for other SRH services.

- Innovation during COVID-19 pandemic

The Centre for COVID-19 Situation Administration (CCSA) was established by the Royal Thai government to update COVID-19 pandemic situation as well as new government policy and intervention to reduce fake news.

#### Quantitative component

The overall survey included 893 participants, as outlined in Table 2, with participants exhibiting certain characteristics. On average, the participants were 25.8 ± 6.7 years old. Half of the participants were in a cohabiting arrangement. About two-thirds of the participants had undergone a COVID-19 test, but only a small proportion of them tested positive. The primary reasons for participant visits were contraception (68.5%), followed by postnatal care (18.4%), and STI services (9.7%).

**Table 2:** Characteristics of clients participated in questionnaire

Variables	Frequency (*n* = 893)
Age	25.8 ± 6.7
Sex	
Female	890 (99.7)
Male	3 (0.3)
Number of school years	13.2 ± 3.0
Marital status	
Single	377 (42.2)
Cohabiting	456 (51.1)
Separated	38 (4.3)
Divorced	16 (1.8)
Widowed	6 (0.7)
Smoking	
No	719 (80.5)
Yes, living with someone who smokes	156 (17.5)
Yes, smoking	11 (1.2)
Smoking and living with someone who smokes	7 (0.8)
Tested for COVID-19	
No	299 (33.5)
Yes	586 (65.6)
Refused to answer	8 (0.9)
Number of pregnancies	
0	301 (33.8)
1	362 (40.7)
2	160 (18)
3	60 (6.7)
4	6 (0.7)
5	1 (0.1)
Number of abortions	
0	776 (87.2)
1	100 (11.2)
2	13 (1.5)
3	1 (0.1)
Number of births	
0	339 (38.1)
1	378 (42.5)
2	150 (16.9)
3	20 (2.2)
4	3 (0.3)
Number of living children	
0	339 (38.1)
1	380 (42.7)
2	149 (16.7)
3	19 (2.1)
4	3 (0.3)
Number of vaginal deliveries	
0	468 (52.6)
1	296 (33.3)
2	108 (12.1)
3	15 (1.7)
Number of Cesarean sections	
0	744 (83.6)
1	113 (12.7)
2	31 (3.5)
3	2 (0.2)
Main reason for current visit	
Abortion care	9 (1)
Antenatal care	5 (0.6)
Delivery care	4 (0.4)
Postnatal care	164 (18.4)
Contraception	612 (68.5)
Violence against women	1 (0.1)
STI	87 (9.7)
Abortion	9 (1)
Other	2 (0.2)
Gestational age (*n* = 27)	
1 st trimester	17 (63)
2nd trimester	6 (22.2)
3rd trimester	4 (14.8)

Data presented as mean ± standard deviation or n (%)

#### Contraception/family planning

Out of the 875 individuals involved in the study, 16.5% were provided with counseling on contraception, while 72.6% received both counseling and contraception (Table 3). Contraceptive implants were the preferred method among women, chosen by 45.8% of clients.

**Table 3:** Contraception/family planning services received by clients

Variable	Frequency
Did client receive contraception services? (*n* = 875)	
No	96 (11)
Counselling	144 (16.5)
Counselling and contraception	635 (72.6)
Contraception option provided (*n* = 779)	
Combined pills	110 (14.1)
Progestin-only pills	18 (2.3)
Combined injectable	4 (0.5)
Progestin injectable	92 (11.8)
Male condom	142 (18.2)
Emergency pill	1 (0.1)
Vasectomy	4 (0.5)
Tubal ligation	35 (4.5)
Contraceptive implant	357 (45.8)
Copper IUD	15 (1.9)
Other natural method	1 (0.1)

Data presented as n (%)

The facility lacked standardized national guidelines. The number of clients at the endline was slightly higher than at the baseline. Certain contraception options, such as the female condom, emergency pill, cycle beads, and vaginal ring, were not in stock. However, both male and female sterilization services were offered at both facilities. Providers did not receive any training on contraceptive methods or adolescent and reproductive health from the baseline to endline period (Table 4).

**Table 4:** Health availability and readiness for family planning services in two facilities in Khon Kaen, Thailand

Variable	Baseline	Endline
Is there a national family planning guideline present in the facility?		
No	2 (100)	2 (100)
Are there any family planning check-list available in the facility?		
No	1 (50)	1 (50)
Yes	1 (50)	1 (50)
How many clients visited facility per month?		
1 st month	83 ± 1.4	110.5 ± 12.0
2nd month	85.5 ± 71.4	129.5 ± 36.1
3rd month	99 ± 46.7	122 ± 35.4
4th month	114 ± 28.3	121.5 ± 21.9
5th month	100 ± 31.1	118 ± 9.9
6th month	104 ± 26.9	111.5 ± 16.3
There are written materials users can take home?		
Yes	1 (50)	1 (50)
No	1 (50)	1 (50)
The facilities stocks contraceptive commodities?		
COCs	2 (100)	2 (100)
POP	1 (50)	1 (50)
Combined EP injectable	0 (0)	0 (0)
Progestin only injectable	2 (100)	2 (100)
Male condom	2 (100)	2 (100)
Female condom	0 (0)	0 (0)
Emergency pill	0 (0)	0 (0)
Cycle beads	0 (0)	0 (0)
Vaginal ring	0 (0)	0 (0)
Contraceptive implant	2 (100)	2 (100)
Copper IUD	2 (100)	2 (100)
Levonorgestrel IUD	2 (100)	2 (100)
Other natural methods	1 (50)	1 (50)
Is male sterilization provided at the facility?		
Yes	2 (100)	2 (100)
Is female sterilization provided at the facility?		
Yes	2 (100)	2 (100)
Human resources		
Have providers received any training in family planning in last 6 months?		
Yes	1 (50)	0 (0)
No	1 (50)	2 (100)

Data present as mean ± standard deviation or n (%)

#### STI services

During the study period, 94 individuals sought STI services. Only 18.8% reported using condoms in their most recent vaginal or anal intercourse. Approximately half of all women underwent STI testing (excluding HIV), with HPV being the most prevalent diagnosis. The services considered most crucial by participants were condom accessibility (44.7%), STI testing (39.4%), and communication with their sexual partner about STIs (28.7%). Notably, 38 participants accessed STI services during the lockdown associated with the COVID-19 pandemic, but they generally found the convenience of receiving these services to be easy or very easy (Table 5).

**Table 5:** Sexual transmitted infection services received by clients

Variable	Frequency (%)
Sex with female partner in the past 3 months (*n* = 94)	
Yes	2 (2.1)
Sex with male partner in the past 3 months (*n* = 94)	
Yes	82 (87.2)
Oral sex with any partner in the past 3 months (*n* = 94)	
Yes	57 (60.6)
Number of male partners	1.2 ± 0.48
Used protection for last oral sex (*n* = 64)	
No	36 (56.3)
Yes	22 (34.4)
Do not know	6 (9.4)
Used protection for vaginal/anal sex (*n* = 69)	
No	56 (81.2)
Yes	13 (18.8)
Do not know	0 (0)
Have been tested for STI other than HIV (*n* = 94)	
No	47 (50)
Yes	44 (46.8)
Do not know	3 (3.2)
Have you ever been told you had an STI other than HIV? (*n* = 44)	
No	9 (20.5)
Yes	33 (75)
Don’t know	2 (4.5)
With which STIs were you diagnosed? (*n* = 33)	
Anal warts	2 (6.1)
Chlamydia	0 (0)
Genital warts (HPV)	15 (45.5)
Gonorrhea	2 (6.1)
Genital herpes	2 (6.1)
Syphilis	9 (27.3)
Trichomoniasis	0 (0)
Mycoplasma genitalium	0 (0)
Unknown	9 (27.3)
What do you think are your most important sexual health needs or concerns? (*n* = 94)	
Access to condom	42 (44.7)
Pre-exposure prophylaxis	13 (13.8)
HIV testing	6 (6.4)
HIV treatment	5 (5.3)
STI testing	37 (39.4)
STI treatment	23 (24.5)
Access to preferred contraception method	18 (19.1)
Abortion care	4 (4.3)
Communication with sexual partner about condoms	13 (13.8)
Communication with sexual partner about HIV	9 (9.6)
Communication with sexual partner about STI	27 (28.7)
Communication with sexual partner about contraception/pregnancy	7 (7.4)
Communication with my health care provider	1 (1.1)
Sexual function	2 (2.1)
Sexual satisfaction	3 (3.2)
Emotional intimacy	2 (2.1)
Sexual coercion	3 (3.2)
Have you been used this STI service? (*n* = 94)	
Yes, last 12 months	29 (30.9)
Yes, more than 1 year ago	9 (9.6)
No	51 (54.3)
Don’t know	5 (5.3)
How difficult was seeing a doctor? (*n* = 38)	
Very difficult	0 (0)
Difficult	2 (5.3)
Moderate	13 (34.2)
Easy	18 (47.4)
Very easy	5 (13.2)
How difficult was getting a lab test for STI? (*n* = 38)	
Very difficult	0 (0)
Difficult	5 (13.2)
Moderate	9 (23.7)
Easy	17 (44.7)
Very easy	5 (13.2)
Not applicable	2 (5.3)
How difficult was getting treatment for STI? (*n* = 38)	
Very difficult	0 (0)
Difficult	3 (7.9)
Moderate	10 (26.3)
Easy	18 (47.4)
Very easy	7 (18.4)

Data present as mean ± standard deviation or n (%)

Both facilities offered HIV counseling and testing services, along with the provision of condoms. Vaccination against HBV and HPV was administered at least in one of the facilities. The target population for these services was limited to adults. All medications, except for spectinomycin, were accessible Table 6 showed that the incidence of cases appeared to be similar during the two measurement periods.

**Table 6:** Health availability and readiness for sexual transmitted infection services in two facilities in Khon Kaen, Thailnd

Variables	Baseline	Endline
Does this facility offer HIV counselling and testing services?		
Yes	2 (100)	2 (100)
Does this facility have HIV rapid test kits (with valid expiration date) in stock today, ready for client testing?		
No	2 (100)	2 (100)
Does this facility have condoms available in this service site today to give to clients receiving services?		
Yes	2 (100)	2 (100)
Does this facility offer diagnosis or treatment of STIs (other than HIV) services?		
Yes	2 (100)	2 (100)
Have you or any provider(s) of STI services received any training in STI diagnosis and treatment in the last two years?		
No	1 (50)	1 (50)
Yes	1 (50)	1 (50)
Are there vaccinations available for		
HAV		
No	2 (100)	2 (100)
HBV		
Yes	2 (100)	2 (100)
HPV		
No	1 (50)	1 (50)
Yes	1 (50)	1 (50)
Target population		
Male adolescent		
No	2 (100)	2 (100)
Female adolescent		
No	2 (100)	2 (100)
Gay men		
No	2 (100)	2 (100)
Transgender		
No	2 (100)	2 (100)
Sex worker		
No	2 (100)	2 (100)
Person with HIV		
No	2 (100)	2 (100)
Adults		
Yes	2 (100)	2 (100)
Drug available for STIs available		
Azithromycin	2 (100)	2 (100)
Erythromycin	2 (100)	2 (100)
Amoxycillin	2 (100)	2 (100)
Metronidazole	2 (100)	2 (100)
Ceftriaxone	2 (100)	2 (100)
Cefixime	2 (100)	2 (100)
Spectinomycin	0 (0)	0 (0)
Number of cases		
1 st month	369.5 ± 38.9	360.0 ± 87.7
2nd month	384.5 ± 125.1	327.5 ± 163.3
3rd month	439.5 ± 74.2	360.5 ± 88.4
4th month	394.5 ± 27.6	381.0 ± 110.3
5th month	436.0 ± 62.2	373.0 ± 89.1
6th month	374.0 ± 89.1	377.0 ± 58.0

Data present as mean ± standard deviation or n (%)

## Discussion

This study showed that disruption occurred primarily in the initial months of the baseline, manifesting as a supply shortage. Despite this challenge, all SRH services were upheld. Both service providers and clients held the perception that the continuity of all SRH services was crucial. Nevertheless, there was a decrease in the number of clients, with some clients opting for long-acting contraceptive methods instead of short-acting ones. When feasible and suitable, certain appointments were deferred. Towards the endline, the impact of these disruptions was less prominent, as a substantial portion of clients had become accustomed to the pandemic and had already been vaccinated.

Both healthcare providers and clients affirmed that people were inclined to postpone childbirth amid the pandemic. This aligns with findings from various international studies published previously [7–9]. The primary reason behind this trend is the fear of immediate risks to both mothers and unborn children. Additional factors, observed during events like the Zika outbreak, encompass economic challenges within families, humanitarian crises, heightened violence, and forced migration [10, 11]. This resulted in increased demand for contraceptive use. Similar finding was observed in the survey from Burkina Faso [8]. Furthermore, our study revealed that nearly half of the clients chose LARC. The option of immediate postpartum insertion of LARC was extended, given its safety profile and the potential to alleviate congestion during postpartum appointments [12].

We found a decrease in number of patients seeking STI testing, especially during the earliest months of the pandemic. It should be noted that in the context of the pandemic, it is advisable to accord precedence to symptomatic patients while postponing preventive screening for STIs, as advocated by the published guideline [13]. This strategic approach is grounded in the rationale that the advantages associated with minimizing exposure to COVID-19 within clinical environments may surpass the adverse consequences stemming from untreated STIs [13, 14]. Therefore, the decline in number of STI testing and treatment was reported in many published studies [15–17]. However, efficiently managing the healthcare infrastructure, particularly through the implementation of measures such as transitioning the registration process to an online platform, medicine delivery by post, holds the potential to mitigate congestion and improve overall operational efficiency. These two processes which originated during COIVD-19 persist afterward.

The pandemic's effects on SRH services involved shortages in supplies and the rescheduling of appointments. A recent scoping review confirmed these adverse effects, leading to restricted access to services [18]. To ensure continuity, a key adaptation was the adoption of telemedicine. VandeVusse et al. [19] observed a swift adoption and growth of telehealth in the US. Telemedicine emerged as a crucial intervention to sustain services amidst the pandemic, particularly for postpartum care and family planning services [20, 21]. The provision of medication through home delivery emerged as a crucial factor during the pandemic, coinciding with the enforcement of lockdown policies and home quarantine measures. Antecedent research has underscored the auspicious potential of this innovative delivery system in addressing pandemic-related challenges. It has been widely embraced and has demonstrated a lack of association with issues related to drug administration [22–24].

Strengths of the study were as follows. First, this is the study using the well-designed method, both quantitative and qualitative, to assess the effect of COVID-19 pandemic on SRH services overtime. Second, the study involves the participation of both healthcare providers and clients, thereby encompassing diverse perspectives comprehensively. Nevertheless, certain limitations are inherent in this research. The restricted number of clients necessitated the extraction of data related to STI services from individuals seeking other SRH services. Additionally, the study exclusively focuses on tertiary hospitals, and therefore, caution should be exercised when extrapolating the findings to different settings.

## Conclusion

This research illustrated that the COVID-19 pandemic exerted a discernible albeit non-significant impact on SRH services. It signifies that the principal referral hospitals in Northeast Thailand maintained service readiness to deliver comprehensive SRH care throughout the pandemic and subsequent recovery period. This resilience can be attributed to various interventions, such as telemedicine and the home delivery of medications, as well as the robust and well-established universal healthcare system in Thailand. It is imperative for Thailand to sustain and enhance its universal healthcare system as a proactive measure to fortify preparedness for potential future pandemics.

## Data Availability

The data will be available upon request as per the WHO policies. Requests for access to data can be sent to alimoa@who.int.

## Abbreviations

SRH: Sexual and reproductive health
WHO: World health organization
HCPs: Healthcare providers
SARA: Service Availability and Readiness Assessment
CCSA: Centre for COVID-19 Situation Administration
UHC: Universal Health Coverage
NHSO: National Health Security Office
OCPs: Oral contraceptive pills
LARC: Long-acting reversible contraception
STI: Sexual transmitted infection
IDIs: In-depth interviews
FGD: Focus group discussion
GBV: Gender-based violence

## References

[1] Tangcharoensathien V, Witthayapipopsakul W, Panichkriangkrai W, Patcharanarumol W, Mills A. Health systems development in Thailand: a solid platform for successful implementation of universal health coverage. Lancet. 2018;391:1205–23. 29397200 10.1016/S0140-6736(18)30198-3

[2] Sothornwit J, Lumbiganon P, Saranrittichai K, Sangkomkamhang U, Singhdaeng T, Jampathong N. Barriers and facilitators to implementing immediate postpartum contraceptive implant programs: a formative implementation research. Int J Womens Health. 2022;14:945–56. 35924095 10.2147/IJWH.S370012 PMC9341331

[3] Triukose S, Nitinawarat S, Satian P, Somboonsavatdee A, Chotikarn P, Thammasanya T, et al. Effects of public health interventions on the epidemiological spread during the first wave of the COVID-19 outbreak in Thailand. PLoS ONE. 2021;16:e0246274. 33606734 10.1371/journal.pone.0246274 PMC7894963

[4] Rajatanavin N, Tuangratananon T, Suphanchaimat R, Tangcharoensathien V. Responding to the COVID-19 second wave in Thailand by diversifying and adapting lessons from the first wave. BMJ Glob Health. 2021;6:e006178. 34285042 10.1136/bmjgh-2021-006178 PMC8295022

[5] Hall KS, Samari G, Garbers S, Casey SE, Diallo DD, Orcutt M, et al. Centring sexual and reproductive health and justice in the global COVID-19 response. Lancet. 2020;395:1175–7. 32278371 10.1016/S0140-6736(20)30801-1 PMC7146687

[6] Kouanda S, Nahyuha Chomi E, Kim C, Jen S, Bahamondes L, Cecatti JG, et al. Health systems analysis and evaluation of the barriers to availability, utilisation and readiness of sexual and reproductive health services in COVID-19-affected areas: a WHO mixed-methods study protocol. BMJ Open. 2022;12:e057810. 35649598 10.1136/bmjopen-2021-057810 PMC9160592

[7] Peng R, Mou W, Xu P. Factors associated with fertility intention among Chinese married youth during the COVID-19 pandemic. Behav Sci (Basel). 2023;13:184. 36829413 10.3390/bs13020184 PMC9951887

[8] Druetz T, Cooper S, Bicaba F, Bila A, Shareck M, Milot D-M, et al. Change in childbearing intention, use of contraception, unwanted pregnancies, and related adverse events during the COVID-19 pandemic: results from a panel study in rural Burkina Faso. PLoS Glob Public Health. 2022;2:e0000174. 36962234 10.1371/journal.pgph.0000174 PMC10021617

[9] Kahn LG, Trasande L, Liu M, Mehta-Lee SS, Brubaker SG, Jacobson MH. Factors associated with changes in pregnancy intention among women who were mothers of young children in New York City following the COVID-19 outbreak. JAMA Netw Open. 2021;4:e2124273. 34524437 10.1001/jamanetworkopen.2021.24273 PMC8444024

[10] Diniz D, Medeiros M, Madeiro A. Brazilian women avoiding pregnancy during Zika epidemic. J Fam Plann Reprod Health Care. 2017;43:80. 28007828 10.1136/jfprhc-2016-101678

[11] Freed B, Hillman S, Shantikumar S, Bick D, Dale J, Gauly J. The impact of disasters on contraception in OECD member countries: a scoping review. Eur J Contracept Reprod Health Care. 2021;26:429–38. 34126834 10.1080/13625187.2021.1934440

[12] Sothornwit J, Kaewrudee S, Lumbiganon P, Pattanittum P, Averbach SH. Immediate versus delayed postpartum insertion of contraceptive implant and IUD for contraception. Cochrane Database Syst Rev. 2022;10:CD011913. 36302159 10.1002/14651858.CD011913.pub3 PMC9612833

[13] Barbee LA, Dombrowski JC, Hermann S, Werth BJ, Ramchandani M, Ocbamichael N, et al. “Sex in the time of COVID”: clinical guidelines for sexually transmitted disease management in an era of social distancing. Sex Transm Dis. 2020;47:427–30. 32541302 10.1097/OLQ.0000000000001194 PMC7448723

[14] Mmeje OO, Coleman JS, Chang T. Unintended consequences of the COVID-19 pandemic on the sexual and reproductive health of youth. J Adolesc Health. 2020;67:326–7. 32690467 10.1016/j.jadohealth.2020.06.019 PMC7367774

[15] Stanford KA, Mason JA, Friedman EE. Trends in STI testing and diagnosis rates during the COVID-19 pandemic at a large urban tertiary care center, and the role of the emergency department in STI care. Front Reprod Health. 2023;5:1082429. 36890799 10.3389/frph.2023.1082429 PMC9986412

[16] Pyra M, Schafer T, Rusie L, Houlberg M, Thompson HM, Hazra A. Temporary changes in STI & HIV testing & diagnoses across different phases of the COVID-19 pandemic, Chicago IL. Front Reprod Health. 2023;5:1072700. 37206577 10.3389/frph.2023.1072700 PMC10188963

[17] Bonett S, Petsis D, Dowshen N, Bauermeister J, Wood SM. The impact of the COVID-19 pandemic on STI/HIV testing among adolescents in a large pediatric primary care network. Sex Transm Dis. 2021;48:e91. 33783411 10.1097/OLQ.0000000000001427 PMC8500357

[18] VanBenschoten H, Kuganantham H, Larsson EC, Endler M, Thorson A, Gemzell-Danielsson K, et al. Impact of the COVID-19 pandemic on access to and utilisation of services for sexual and reproductive health: a scoping review. BMJ Glob Health. 2022;7:e009594. 36202429 10.1136/bmjgh-2022-009594 PMC9539651

[19] VandeVusse A, Castillo PW, Kirstein M, Mueller J, Kavanaugh M. Disruptions and opportunities in sexual and reproductive health care: how COVID-19 impacted service provision in three US states. Perspect Sex Reprod Health. 2022;54:188. 36351551 10.1363/psrh.12213 PMC9878085

[20] Sothornwit J, Kaewrudee S, Somboonporn W, Seanbon O, Ngamjarus C. Implementing the individualized postpartum care with telemedicine during the COVID-19 pandemic at tertiary hospital in Thailand. Heliyon. 2023;9:e16242. 37229160 10.1016/j.heliyon.2023.e16242 PMC10182597

[21] Phianphitthayakul O-A, Li J, Rongkapich R, Karroon P, Vatrasresth J, Jaisamrarn U, et al. Client experiences with telehealth using LINE for consultation and assessment of adverse effects of contraceptive implants during the COVID-19 pandemic in Thailand. Digit Health. 2023;9:20552076231203876. 37780063 10.1177/20552076231203877 PMC10540598

[22] Chaomuang N, Dede AJO, Saokaew S, Umnuaypornlert A. Effects of home drug delivery on drug-related problems: preliminary evidence for improved patient outcomes during the COVID-19 pandemic in Thailand. J Am Pharm Assoc. 2003;2022(62):1206-1213.e3. 10.1016/j.japh.2022.01.015 PMC8782741 35151582

[23] PeláezBejarano A, Villar Santos P, de LA Robustillo-Cortés M, Sánchez Gómez E, Santos Rubio MD. Implementation of a novel home delivery service during pandemic. Eur J Hosp Pharm. 2021;28:e120–3. 33115800 10.1136/ejhpharm-2020-002500 PMC8640397

[24] Brey Z, Mash R, Goliath C, Roman D. Home delivery of medication during Coronavirus disease 2019, Cape Town, South Africa: short report. Afr J Prim Health Care Fam Med. 2020;12:2449. 32501022 10.4102/phcfm.v12i1.2449 PMC7284162

